# Cold Plasma Activity Against Biofilm Formation of Prosthetic Joint Infection Pathogens

**DOI:** 10.3390/pathogens14010010

**Published:** 2024-12-28

**Authors:** Christopher Spiegel, Débora C. Coraça-Huber, Michael Nogler, Rohit Arora, David Putzer

**Affiliations:** 1 Research Laboratory for Biofilms and Implant Associated Infections (BIOFILM LAB), Experimental Orthopaedics, University Hospital for Orthopaedics and Traumatology, Medical University of Innsbruck, Müllerstraße 44, 6020 Innsbruck, Austria; debora.coraca-huber@i-med.ac.at; 2 Department of Orthopaedics and Traumatology, Medical University of Innsbruck, Anichstraße 35, 6020 Innsbruck, Austria; michael.nogler@tirol-kliniken.at (M.N.); rohit.arora@tirol-kliniken.at (R.A.); david.putzer@i-med.ac.at (D.P.)

**Keywords:** cold atmospheric plasma, biofilm, prosthetic joint infection, staphylococci, orthopedics

## Abstract

Periprosthetic joint infections occur in 1–2% of all patients undergoing prosthetic joint surgeries. Although strong efforts have been made to reduce infection rates, conventional therapies like one- or two-stage revisions have failed to lower the infection rates. Cold atmospheric plasma (CAP) has shown promising results in reducing bacterial loads on surfaces. In this study, we aimed to investigate the ability of CAP to reduce the bacterial load on metal surfaces with varying distances and different plasma compositions below a temperature suitable for in vivo applications. Methods: Biofilm was formed with *Staphylococcus aureus* ATCC 29213 and *Staphylococcus epidermidis* ATCC 12228 cultures on TMZF discs. Plasma treatments using air plasma and argon plasma were conducted on discs containing the established biofilm while the temperature was measured. During the experiments, the duration and the distance of plasma application varied. Afterwards, colony-forming units were counted. Results: The results of this study showed that air and argon plasma could be considered for applications during surgeries at a 1 cm distance. While air plasma showed the highest efficiency in CFU reduction, the temperature generation due to the presence of oxygen poses a limitation concerning the duration of application. The use of argon as a plasma generator does not show the temperature limitation in correlation to exposure time. The use of air plasma with a distance of 1 cm to the application site and an exposure time of 5 s showed the most effective bacterial reduction while not exceeding tissue-damaging temperatures.

## 1. Introduction

Bacterial infections pose significant challenges in medical treatment and can be life-threatening in severe cases [1,2]. Antibiotic therapies are widely regarded as a key method for treating these infections [3]. However, with the global rise in antibiotic use, unregulated usage has become inevitable. This misuse has led to widespread bacterial resistance, making it one of the most serious threats to human health [4]. A potential solution lies in identifying new bactericidal approaches that bypass antibiotic resistance. Cold atmospheric plasma (CAP) has shown promise in this regard [5].

Implant-related infections occur in approximately 1–2% of all implantation surgeries, with prosthetic joint infections (PJIs) in total hip replacements leading to treatment costs ranging from $40,000 to $160,000 per case [6,7]. The 5-year mortality rate associated with PJIs can reach up to 21% [8], underscoring the significant burden these biofilm-associated infections impose on both patients and healthcare systems. Numerous strategies have been explored to modify implant surfaces, aiming to improve osseointegration while minimizing the risk of infection [9,10,11]. Also, prior to clinical use as indwelling medical devices, all biomaterials must undergo thorough sterilization [12].

PJIs remain prevalent due to factors such as the increasing complexity of surgeries and the durability of implants. One critical aspect is the loss of cement bond strength, which can lead to mechanical loosening and biofilm formation [13,14]. The degradation of cement strength over time not only compromises implant stability but also creates a favorable environment for bacterial adhesion and biofilm formation.

Biofilm formation is particularly problematic as it protects bacteria from systemic antibiotics and host immune responses. While the ica operon is well known for its role in biofilm formation, alternative mechanisms such as Embp, AtlE, Bap, fbe, Esp, and lpxtG have also been identified in *Staphylococcus* species, emphasizing the complexity of biofilm-related infections [15]. Additionally, traditional bone cement materials, often used in TJR, are limited in their antimicrobial capabilities. Improved mechanical properties and antimicrobial functionality are crucial to addressing these issues.

The TiMo12Zr6Fe2 (TMZF) alloy is a promising material for orthopedic applications due to its excellent mechanical properties, biocompatibility, and corrosion resistance. Its elastic modulus closely matches that of natural bone, reducing the risk of stress shielding and promoting implant longevity [16]. TMZF supports the adhesion, proliferation, and matrix production of osteoblasts and fibroblasts, facilitating effective osseointegration [17]. These properties make it particularly suitable for joint replacements, spinal implants, and dental prostheses, where long-term stability is essential [18]. Compared to traditional materials like stainless steel and cobalt–chromium alloys, TMZF offers improved biocompatibility and mechanical compatibility, enhancing patient outcomes [19]. Furthermore, its resistance to corrosion minimizes adverse reactions associated with ion release over time [16].

There have already been many approaches made to implement cold plasma as a therapeutic tool against peri-implantitis. Cold plasma has shown bone-strengthening properties in enhancing bone formation around an implant, which is also called osseointegration. Therefore, cold plasma can be considered as a helpful tool against peri-implantitis [20]. To enhance the foreign body reaction, which is essential for osseointegration, reconditioning the implant’s surface by removing disguising carbon layers to the immune system can be useful to reinstate osseointegration [21]. In 2018, Yang et al. were able to show that a cold plasma treatment of peri-implantitis implants reduced the existing carbon layer, sterilized the implant surface, and enhanced osseointegration [22]. Another important factor for using cold plasma for peri-implantitis treatment is the sterilization of the implant surface and the peri-implant tissue. In a study where peri-implantitis pathogens were used, cold atmospheric plasma treatment was able to reduce the bacterial count by three log10 and reduce microbial metabolic activity by 95–98% while enhancing fibroblast adhesion [23].

Biofilms are complex microbial communities embedded in a matrix of extracellular polymeric substances (EPSs) like polysaccharides, proteins, eDNA, and phospholipids [5,24]. Bacteria primarily grow as biofilms due to their protective benefits, enabling survival in harsh environments [25]. Cold plasma acts on biofilms similarly to planktonic bacteria, causing damage to cell membranes, proteins, and nucleic acids [26]. Additionally, cold plasma disrupts EPSs and interferes with quorum sensing (QS).

EPSs facilitate strong cell–cell interactions within biofilms, supporting cell communication, gene transfer, and micro-consortia formation [25]. CAP causes oxidative damage to EPS components, including lipid peroxidation, protein degradation, and carbohydrate bond breakage, leading to the disintegration of the biofilm structure [27,28]. This breakdown reduces biofilm adhesion, potentially converting biofilm bacteria into their planktonic form, allowing for more effective disinfection [29]. Quorum sensing, an intercellular signaling system regulating biofilm formation and virulence, is also disrupted by cold plasma as it affects the synthesis of and degrades the signaling molecules (autoinducers) that mediate communication between bacteria [30,31].

The study aimed to investigate the ability of CAP to reduce the bacterial load on metal surfaces with varying distances and different plasma compositions below a temperature suitable for in vivo applications.

## 2. Materials and Methods

### 2.1. Biofilm Formation

Biofilm formation was performed on TMZF discs. *Staphylococcus aureus* ATCC 29213 and *Staphylococcus epidermidis* ATCC 12228 were used for biofilm formation [32,33,34]. At first, an overnight culture was prepared using each strain by taking three colonies as an inoculum from a prepared plate agar culture. The overnight culture was inoculated in 2 mL of Müller–Hinton broth (MH broth). In previous studies, MH broth showed sufficient biofilm formation results for *Staphylococcus aureus* ATCC 29213 [35]. After overnight incubation, the pre-culture was diluted with MH broth to a value of 0.4–0.6 MF units with a McFarland Densitometer (Den1B, Grant Instruments, Royston, UK). The discs were placed in each well of a 24-well plate and filled with 200 µL diluted pre-culture. For each treatment, three discs were used as parallels. After 24 h of incubation at 37 °C in a moisture chamber, the discs were ready to be treated with cold plasma.

### 2.2. Plasma Treatment

The cold plasma treatment was performed with the micro cold plasma MCP500-1 machine (INOCON TECHNOLOGIE 4800, Attnang-Pucheim, Austria). Micro cold plasma MCP500-1 generates its plasma between a non-melting stainless steel electrode and the nozzle by means of an arc. The process gases used were compressed air, argon gas, and a mixture of argon and compressed air. The flow rate was approximately 22 L/min for compressed air and approximately 11 L/min for argon and the argon–compressed air mixture.

**Figure 1:**
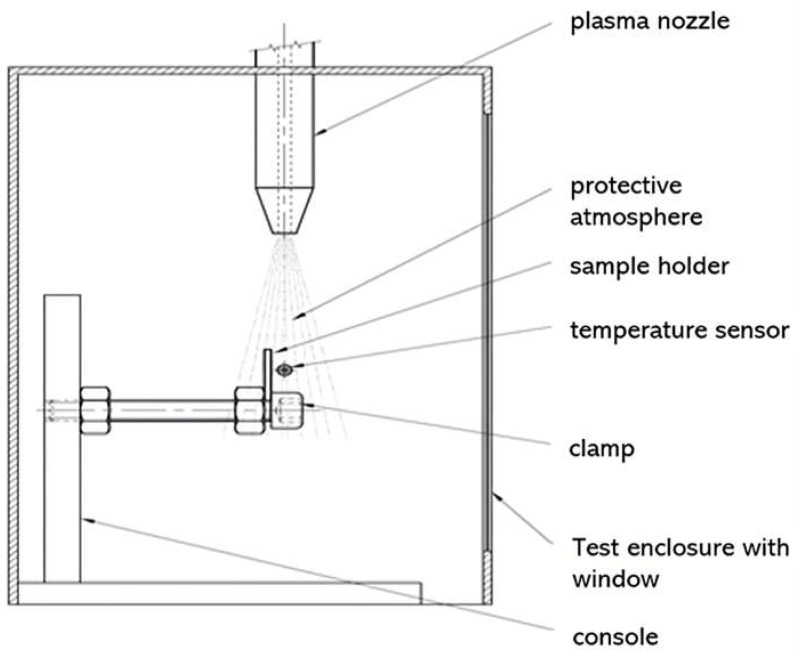
Plasma treatment within the cold plasma chamber. The chamber guarantees constant controllable experiments using fixed distance control and consistent temperature measurements.

For plasma application and temperature censoring, a safety cage was used around the cold plasma device (Figure 1). The cold plasma exits at the tip of the plasma nozzle, while the treated sample is kept in place by a clamp. At the application site, a temperature sensor is also held in place to perform consistent temperature measurements during plasma application (Figure 1).

The experiments were conducted in different stages. At first, we tested for the most effective application distance during different exposure times with air plasma. Air plasma was the plasma with the highest O_2_ content and the highest temperature to be expected. During the first experiments, *Staphylococcus aureus* ATCC 29213 was used for biofilm formation in the pre-study. Afterwards, the experiments were conducted with a distance of 1 cm and different exposure times with air plasma, argon plasma, and an air–argon plasma mixture. *Staphylococcus epidermidis* ATCC 12228 was used for these experiments. Temperatures were monitored during the experiments, and colony-forming units were counted.

### 2.3. Colony-Forming Unit Counting After Plasma Treatment

Immediately after the plasma treatment, the surface temperature was measured. Following the plasma treatment, the discs were washed in PBS and sonicated in 2 mL of PBS for 1 min at high intensity (BANDELIN BactoSonic, Berlin, Germany). Afterwards, the sonicated PBS samples were properly diluted, plated on Müller–Hinton agar plates, and the CFUs were counted after 24 h of incubation at 37 °C.

### 2.4. Statistics

We used GraphPad Prism 9 for our statistical analysis. We calculated the average CFUs and standard deviations, which we show in bar graphs. For gene expression, we determined the fold change using delta-delta CT values for each replicate and then calculated standard deviations after averaging the data for each gene and condition. The results are presented in bar graphs that include the standard deviations.

## 3. Results

### 3.1. Preliminary Study to Evaluate Effective Distance to Temperature and Time Exposure Settings

**Figure 2:**
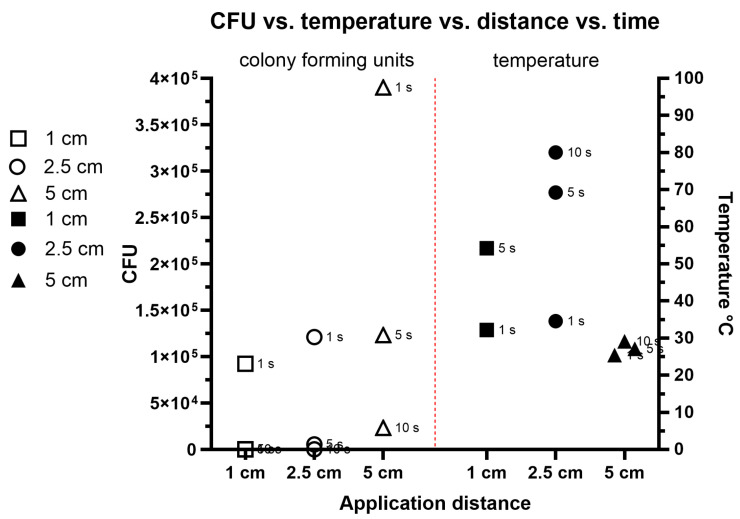
Preliminary study to evaluate the influence of application distance and exposure time on colony-forming units and temperature development. On the left side with white markers are the colony-forming units, with dependency of application distance and exposure time shown. On the right side of the figure, the temperature during the cold plasma treatment with dependency of distance and exposure time is shown. The treatment during this experiment was conducted with air plasma.

The preliminary study showed an effective bacterial count reduction in all distances and application times conducted (Figure 2). The control showed a bacterial growth of 5.2 × 10^8^ CFU. At the 1 cm application distance, after 1 s of exposure there were 9.2 × 10^4^ CFUs present, whereas after 5 s no bacteria were found alive. The temperatures at this distance were 32.1 °C after 1 s of exposure and 52.1 °C after 5 s of exposure. At a distance of 2.5 cm, the bacteria grew after 1 s of exposure up to 1.2 × 10^5^ CFU, after 5 s of treatment to 5.4 × 10^3^, and after 10 s of treatment no bacteria were able to be detected. During these treatments, the temperatures were 34.5 °C after 1 s, 69.1 °C after 5 s, and 80 °C after 10 s. At a distance of 5 cm and after 1 s of treatment, 3.9 × 10^5^ CFUs were detected, whereas after 5 s and 10 s of treatment, 1.2 × 10^5^ and 2.3 × 10^4^ CFUs were detected, respectively. The temperatures during these treatments were 25.1 °C for 1 s of application, 27.1 °C after 5 s of treatment, and at 29.1 °C after 10 s.

After the first experiments, the application distance of 1 cm showed the most efficient antibacterial effect. Therefore, all following experiments with different uses of gas for cold plasma generation were conducted at a 1 cm application distance and different exposure rates.

### 3.2. Influence of Exposure Time on Temperature and Bacterial Growth with Different Plasma Types

#### 3.2.1. Air Plasma

**Figure 3:**
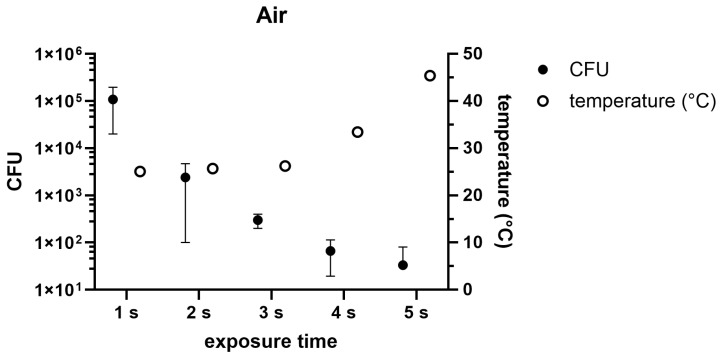
Colony-forming units under the influence of cold air plasma are marked as black dots at a 1 cm application distance at exposure times from 1 to 5 s. During these procedures, temperatures were measured, marked in white.

The use of air for cold plasma generation showed an antimicrobial effect at a distance of 1 cm (Figure 3). After 1 s of treatment, 1.08 × 10^5^ CFUs (SD: 8.8 × 10^4^ CFUs) were counted. After 2 s 2.4 × 10^4^ CFUs (SD: 2.3 × 10^3^ CFUs), after 3 s 3 × 10^2^ CFUs (SD: 3 × 10^2^ CFUs), after 4 s of treatment 6.6 × 10^1^ CFUs (SD: 4.7 × 10^1^ CFUs) and after 5 s 3.3 × 10^1^ (SD: 4.7 × 10^1^ CFUs) CFUs were detected. During this experiment, the following values were measured from 1 to 5 s of incubation: 25 °C, 25.7 °C, 26.2 °C, 33.4 °C and 45.4 °C.

#### 3.2.2. Argon Plasma

**Figure 4:**
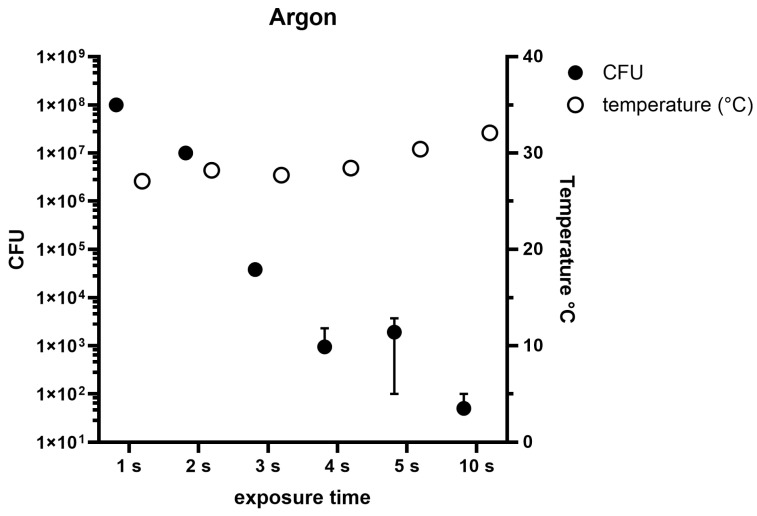
Colony-forming units under the influence of argon cold plasma are marked as black dots at a 1 cm application distance at exposure times from 1, 2, 3, 4, 5, to 10 s. During these procedures temperatures were measured, marked in white.

The use of Argon as a cold plasma gas showed after 1 s of exposure time 1 × 10^8^ CFUs (SD: 0 CFUs) (Figure 4). After 2 s, the CFUs were reduced to 1 × 10^7^ counts (SD: 0 CFUs). The CFU counts after 3 s of cold plasma treatment showed 3.8 × 10^4^ CFUs (SD: 8 × 10^3^ CFUs). After 4 s of treatment, 9.43 × 10^2^ CFUs (SD: 1.3 × 10^3^ CFUs) were counted. After 5 s of treatment 1.9 × 10^3^ CFUs (SD: 1.8 × 10^3^ CFUs) were found. At the last treatment duration of 10 s, we found 5 × 10^1^ CFUs (SD: 5 × 10^1^ CFUs). During the experiment, the following temperatures were recorded for the exposure times of 1–10 s: 27 °C (1 s), 28.2 °C (2 s), 27.7 °C (3 s), 28.4 °C (4 s), 30.4 °C (5 s), and 32.1 °C (10 s).

#### 3.2.3. Mixed Plasma

**Figure 5:**
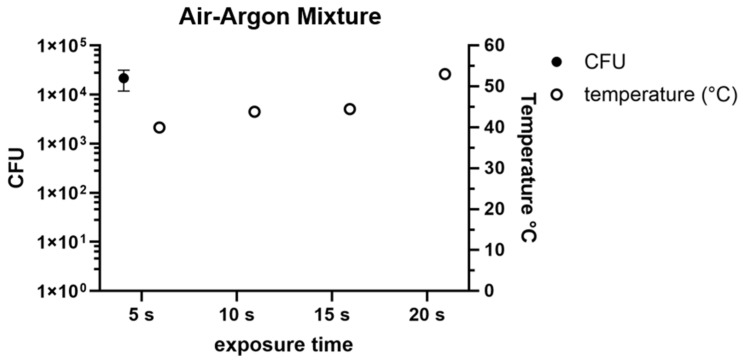
Colony-forming units under the influence of argon cold plasma are marked as black dots at a 1 cm application distance at exposure times from 5, 10, 15, and 20 s. During these procedures, temperatures were measured, marked in white.

The combination of air and argon for cold plasma generation showed a bacterial growth of 2.1 × 10^5^ CFUs after 5 s of exposure (SD: 9.8 × 10^3^ CFUs) (Figure 5). Respectively, 10, 15, and 20 s of treatment all showed no growth of bacteria. The temperatures recorded during these treatments were 39.9 °C for 5 s, 43.8 °C for 10 s, 44.4 °C for 15 s, and 53 °C for 20 s.

## 4. Discussion

Cold plasma technology is revolutionizing surgical practices through its diverse applications and unique properties. A partially ionized gas at near-room temperature, cold plasma is rich in reactive oxygen and nitrogen species, making it highly effective for sterilization and wound management [36]. Its antimicrobial properties allow it to kill bacteria, viruses, and fungi, significantly reducing the risk of post-surgical infections [37]. In cancer surgeries, it shows promise in targeting malignant cells with precision while sparing healthy tissue, enhancing the chances of complete tumor resection [38]. Dermatological and cosmetic surgeries also benefit from cold plasma’s ability to remodel skin and minimize scarring [39]. This technology offers a minimally invasive approach, as it operates at near-room temperature and avoids thermal damage to tissues. As research progresses, cold plasma’s potential to improve surgical outcomes is becoming increasingly evident. By integrating cold plasma into standard medical practice, healthcare systems can achieve safer and more efficient surgical interventions. The preliminary study (Figure 2) showed that an increase in distance reduced the antibacterial effectiveness of the cold air plasma. After 1 s of exposure, an increase in distance led to a higher CFU count in comparison between distances of 1, 2.5, and 5 cm. Furthermore, a longer exposure time showed more effect on CFU reduction in comparison to samples after 5 and 10 s of exposure. In previous studies cold plasma treatment showed promising results on reducing bacterial counts on implant surfaces [40]. Zeng et al. have shown that a biofilm decontamination on titanium lead to a 10 fold reduction of bacterial cell counts after 30 s of cold atmospheric plasma treatment [41]. The authors did not measure the involvement of temperature during their study. Ziuzina et al. were able to examine that *Staphylococcus aureus* showed the highest resilience towards cold plasma treatment. After 300 s of treatment 10^2^ CFUs was detected [42]. In a previously conducted study of Dahle et al. [43] the authors were able to show a correlation of bacterial reduction effectiveness towards time and distance [39]. Using *Staphylococcus aureus* for biofilm formation, after 10 s of treatment at 1 mm distance a reduction of 3 logs CFUs was reached. The results of our study showed a higher effectiveness of cold plasma than previously examined. We showed by using air as plasma a reduction of around 5 logs CFUs after 5 s of treatment. Several mechanisms by which cold plasma inhibits bacteria have been identified in previous studies which include oxidation and perforation of the cell membrane, protein degradation and modification, nucleic acid modification and chain-breaking, disruption of extracellular polymeric substances (EPSs), and interference with quorum sensing (QS) in biofilms [25,41,44]. Therefore, a longer exposure to cold plasma leads to a stronger bacterial cell destruction. The increase in temperature of over 50 °C during longer exposure times and the highest efficiency of CFUs reduction at a 1 cm distance led to further investigations at a fixed 1 cm distance and varying exposure times, which were conducted under the 50 °C temperature limit. The application distance of 1 cm seems also feasible to be applied during a surgery.

Thermal cellular bone necrosis occurs with prolonged exposure to temperatures higher than 56 °C as it induces collagen denaturation as well as damage to surrounding soft tissue, bone marrow and cells crucial for post-operative healing is observed [45]. At higher thermal intervals (temperatures of 48–60 °C) the required exposure time to attain irrecoverable thermal damage is a few seconds [46].

In the following experiments, the distance was constant at 1 cm whilst the exposure times varied. The use of air plasma (Figure 3) showed an effective CFU reduction after 5 s. The temperatures during these treatments did not rise over the 50 °C limit. Therefore, a cold air plasma treatment of 5 s at a 1 cm distance followed by a wash step during the surgery can be applied to reduce bacterial contaminations. At a 1 cm distance and exposure times of 1, 2, 3, 4, 5, and 10 s, argon plasma showed the most effective bacterial count reduction at 10 s. While air plasma showed a 2 log lower CFU count already after 5 s of treatment, argon plasma still had 1.9 × 10^3^ CFUs after 5 s of treatment (Figure 4). During the treatment with argon plasma, the temperatures ranged between 27 and 32 °C. As the temperatures are lower during application, argon plasma could be considered as the more suitable option for use during surgery. However, with the lower CFU reduction effectiveness, air plasma still could be considered as the better option. The combination of air and argon for plasma generation showed stable temperature developments during the treatments of 5, 10, 15, and 20 s of exposure, of 39 °C to 53 °C (Figure 5). These times were chosen as the exposure of 5 s still showed a high CFU count of 2.1 × 10^5^ CFU. Compared to the use of pure air plasma or pure argon plasma, the combination of air and argon showed the least effective reduction of bacterial counts. However, the longer exposure times showed no CFU counts. The mixture of air and argon could be considered as the least effective option for CFU reduction as the temperatures generated during the treatments reached the 50 °C limit. This study is limited by in vitro experiments. In vivo experiments would give deeper insights into the effectiveness and applicability of CAP as a valuable tool during surgeries. For future experiments, the use of clinical strains and methicillin-resistant *Staphylococcus aureus* strains and polymicrobial biofilms is recommended. To understand the mechanisms and to develop new alternatives towards PJI therapies, further research needs to be conducted.

## 5. Conclusions

The results of this study showed that air and argon plasma could be considered for applications during surgeries at a 1 cm distance. While air plasma showed the highest efficiency in CFU reduction, the temperature generation due to the presence of oxygen poses a limitation in the application duration. The use of argon as a plasma generator does not show the limitation in temperature in correlation with exposure time. However, as time is an important factor during surgeries, longer exposure times are unwanted. The same factor could be applied to the air–argon mixture, which also operates at the temperature limits to show effective bacterial count reduction. Therefore, we suggest the use of air plasma with a distance of 1 cm to the application site and an exposure time of 5 s. Due to the reduced operating temperatures, it might be a suitable method for reducing bacterial activity within the surgical site of the patient. The use of cold plasma should be combined with antibiotic therapy and additional sterilization procedures at the surgery site of the patient.

## Data Availability

All data are presented in the publication.
